# Dataset on the effect of gamification elements on learning effectiveness among Vietnamese students

**DOI:** 10.1016/j.dib.2023.109734

**Published:** 2023-10-28

**Authors:** Bang Nguyen-Viet, Bac Nguyen-Viet, Cuong Nguyen-Duy

**Affiliations:** University of Economics Ho Chi Minh City, Vietnam

**Keywords:** Gamification, Learning effectiveness, Intrinsic motivation, Satisfaction, Engagement, Gamified classes

## Abstract

The enhancement of learning effectiveness among university and graduate students is a continual and perplexing question for educational institutions. With the advancement of technology, educators are exploring and implementing diverse learning methods. In this current climate, gamification has emerged as an innovative and popular approach to education. Therefore, it is crucial to thoroughly examine the impact gamification has on the learning effectiveness of university and graduate students. The data also includes demographics of the participants. The questionnaire-based data was collected from previous studies with university and graduate students in Vietnam. The study used Excel and SmartPls 4.0 software to analyze data.. This research can serve as a resource for educational managers, lecturers, and universities to comprehend the effect of gamification on learning effectiveness. It can also aid researchers in the field to explore further the impact of gamification elements and their mediating roles, ultimately enhancing the learning effectiveness of university and graduate students.

Specifications Table

**Table:** 

Subject	Social Sciences
Specific subject area	Education
Type of data	Tables and figures and raw attached dataset
How data were acquired	Survey with the development of the questionnaire, which was modified from prior studies.
Data format	Raw data and analyzed statistical data through Excel and SmartPLS 4.0 software
Parameters for data collection	Respondents are students and graduate students at universities in the northern and southern regions of Vietnam.
Description of data collection	Data was collected by random method. The questionnaire was broadcast online to students and graduate students at 10 universities in Vietnam. The dataset consists of 255 valid responses.
Data source location	City/Town/Region: Universities in Ho Chi Minh City and Hanoi
Data accessibility	Data and the questionnaire used in the survey are available in this article. https://data.mendeley.com/datasets/6n8brzcg7t/3

## 1 Value of the Data

• The dataset at our disposal has the potential to shed light on the intricacies of decision-making pertaining to the incorporation of gamification elements in the educational framework.
• The dataset is useful to investigate how gamification elements can optimally stimulate learning by cultivating student satisfaction, overall engagement, and intrinsic motivation across students at both the undergraduate and graduate levels.
• The valuable dataset at hand is an exceptional resource for those researchers who wish to delve into the vast realm of gamification elements, specifically those which fuel challenge, competition, and enjoyment, and how they significantly interplay with learning effectiveness through various aspects, ranging from satisfaction to engagement, and intrinsic motivation.
• It is imperative to note that the dataset is equally beneficial not only for educational business managers and professors but also for universities that thread on a path of immense learning effectiveness for both their undergraduate and graduate students.
• The data can be used for future research to analyze other potential elements influencing learning effectiveness, engagement, satisfaction, and intrinsic motivation at different educational levels and in different countries for comparison.
• This particular set of data holds the potential to furnish valuable insights regarding the reliability and validity of adaptive instruments.

## 2 Objective

This dataset was generated to provide the input data to estimate the impact of gamification elements to learning effectiveness through satisfaction, engagement and intrinsic motivation. The methodology has been applied to monitor college students and graduate students in the post-Covid-19 context in Vietnam.

## 3 Data Description

In the repository, we provide questionnaire files in English used to investigate latent and dependent variables. Furthermore, we also provide a data file that is the result after surveying the gamification factors affecting learning effectiveness through satisfaction, engagement and intrinsic motivation in Vietnam.

Ho Chi Minh City and Hanoi are two bustling cities in Vietnam that have been studied to understand the effects of gamification on education. These cities were chosen due to their large and established universities, making it easier to collect data. The focus was on college students who were taking courses on gamification, as many Vietnamese undergraduates are not accustomed to using technology and find it difficult to see its potential for enhancing their learning experience. As a result, only college students and researchers were included in the study, and a Vietnamese version of the questionnaire was used.

255 responses were considered valid after screening for duplicates and consistent responses. The majority of participants were female (56.9%) and between 22 and 26 years old (34.1%). Graduate students made up 29% of the sample, while the remaining 71% were undergraduate students. The survey was conducted in Ho Chi Minh City and Hanoi, with 69.8% and 30.2% of participants from these respective cities (Table 1, Table 2, Table 3).

**Table 1:** Demographic profile of respondents.

Measure	Items	Frequency	%
Gender	Male	110	43.1
	Female	145	56.9
Area	Ha Noi	77	30.2
	Ho Chi Minh	178	69.8
Age	From 18 years to 22 years	67	26.3
	From 22 years to 26 years	87	34.1
	From 26 years to 30 years	64	25.1
	Over 30 years	37	14.5
Education	Student	181	71
	MA Student	74	29

**Table 2:** Constructs with items and reliability and validity.

Constructs with Items	OL	CA	CR	AVE
**Competitveness (Adopted from**[3]**)**				
CO1: I feel competitive with other students when using gaming platforms	0.855	0.783	0.873	0.698
CO2: Gaming Platforms provide a competition system when I take a quiz	0.881			
CO3: I want to get the highest rating when using the gaming platforms	0.765			
**Enjoyment (Adopted from**[3]**)**				
ENJ1: I enjoy using games in the classes	0.884	0.908	0.936	0.784
ENJ2: I feel comfortable with the game-based classes	0.885			
ENJ3: I'm enthusiastic about game-based classes when carrying out the learning process	0.896			
ENJ4: I feel happy when I spend time with games for learning	0.876			
**Challenge (Adopted from**[3]**)**				
CH1: Gaming platforms indicate the number of questions to be answered	0.726	0.788	0.862	0.610
CH2: Gaming platforms provide video or image aids to help solve questions	0.775			
CH3: Gaming platforms provide a time limit for answering questions	0.786			
CH4: I feel challenged when competing in the game-based classes	0.833			
**Intrinsic Motivation (Adopted from**[4]**)**				
IM1: I enjoyed the game-based classes very much.	0.871	0.860	0.904	0.704
IM2: Game-based classes was fun to take	0.847			
IM3: I thought game-based classes was boring (reversed).	0.742			
IM4: I would describe the game-based classes as very interesting.	0.889			
**Satisfaction (Adopted from**[3]**)**				
SA1: Overall, I am satisfied with game-based learning	0.891	0.838	0.903	0.755
SA2: Gaming platforms that I used in classes have met my expectations	0.860			
SA3: I am delighted with the experience I get when using gaming platforms in classes	0.856			
**Engagement (Adopted from**[5]**)**				
ENG1: Whenever I have to use games in classes, I usually use	0.750	0.875	0.909	0.668
ENG2: I am passionate about the games in classes	0.875			
ENG3: I love the games in classes	0.854			
ENG5: I am excited when using the games in classes	0.822			
ENG6: I am proud of using the games in classes.	0.779			
**Effectiveness Learning (Adopted from**[6]**)**				
EP1: Game-based learning improves my grade in the class	0.899	0.860	0.915	0.782
EP2: Applying game-based learning encourages me to continue learning on the gamified platform by myself	0.856			
EP3: Use of the gaming platforms have improved my overall learning performance	0.897			

*Note:* OL: Outer Loading, CR: Composite Reliability, AVE: Average Variance Extracted.

**Table 3:** Results of test for discriminant validity.

	Mean	CH	CO	ENG	ENJ	LE	IM	SA
**CH**	4.159	**0.781**						
**CO**	4.201	0.699	**0.835**					
**ENG**	3.821	0.671	0.523	**0.817**				
**ENJ**	4.295	0.727	0.673	0.649	**0.885**			
**LE**	3.839	0.585	0.444	0.697	0.477	**0.884**		
**IM**	4.130	0.588	0.546	0.676	0.717	0.605	**0.839**	
**SA**	4.033	0.610	0.458	0.744	0.608	0.637	0.660	**0.869**

*Note:* The bold diagonal elements are the square root of the variance shared between the constructs and their measures; off diagonal elements are the correlations among constructs.

To ensure the reliability of the research, Cronbach's alpha and composite reliability (CR) were calculated, both of which showed that the scales were reliable. The average extracted variance (AVE) met the threshold of 0.5, indicating convergence [1]. Additionally, the outer loading of the measurement model for all constructs was over 0.708, indicating internal consistency [2]. Discriminant validity was confirmed by comparing the square root of a construct's AVE with its bivariate correlation with other constructs in the model. The square root of the AVE ranged from 0.781 to 0.885, indicating adequate discriminability.

## 4 Experimental Design, Materials and Methods

### 4.1 Questionnaire development

The scales used to measure the constructs were adapted from prior studies, in which the scales had already been proven to be valid and reliable. The wording of the scales was slightly modified to suit the research context. The questionnaire designed for this study was originally drafted in English, translated into Vietnamese, then back to English by two native Vietnamese speakers to ensure it corresponded with the English version. A back-translation method was followed to accurately depict the exact meaning of the questionnaire in the target language [7]. The concept of "Competitive" scale with 03 observed variables is inherited by Wirani et al. [3]; “Enjoyment” has 04 observed variables inherited by Wirani et al. [3]; “Challenge” has 04 observed variables inherited by Wirani et al. [3]; “Satisfaction” has 03 observed variables inherited by Wirani et al. [3]; “Engagement” has 05 observed variables inherited by Kamboj et al. [5]; “Intrinsic Motivation” has 04 observed variables inherited by Torbergsen et al. [4]; “Effectiveness Learning” has 03 observed variables inherited by Damnjanovic et al. [6]. Those measures use five-point Likert scale, from 5 (strongly agree) to 1 (strongly disagree).

To ensure its effectiveness, the questionnaire underwent a pre-test phase that involved gathering feedback from 15 people who have used gamification in education and they are professionals in marketing sector. This process aimed to assess the wording, sequencing, and completeness of the questionnaire. In response to feedback from the participants, modifications were made to the questionnaire's sequence, ambiguous questions were removed, and certain phrasing was adjusted. Consequently, this feedback contributed to enhancing the questionnaire's clarity, relevance, and overall consistency, as highlighted by Hair et al. in their 2010 study.

### 4.2 Questionnaire content

The questionnaire included 26 items and It takes about 6 min to complete. The questionnaire consists of 2 parts. The first part consists of questions asking about perceived personal point of view of each question. The second part consist of questions on demographic profile of the respondents. Demographic information including Gender, Area, Age, Education.

### 4.3 Data collection

The study conducted a field study using online surveys. The researchers sent out project information and consent forms to students through teachers’ teaching classes and student groups on social networking platforms such as Facebook and Zalo. The questionnaire drew on a literature review and consultation with experts to ensure the validity of the data collection tool. To avoid response bias, a native speaker distributed the final version of the survey to respondents and offered the option to complete it online or in paper format [8]. Respondents who had interacted with gamification in courses continued to answer follow-up questions, while those who had not were asked to withdraw from the survey. The researchers used Google Forms to create the survey tool and created a link to the questionnaire. Respondents were required to provide an email address to ensure the validity of the questionnaire. Morever, there are no correct answers and that all responses will be kept anonymous [8]. Futher, to minimize common method bias, we used reverse-worded items [9]. We examined the responses to IM3 (I thought game-based classes were boring) as a means to identify substandard answer sheets. This process aimed to pinpoint and address any inconsistencies or unreliable answers provided by the survey participants. It ensures that the data used for analysis accurately represents the participants' genuine attitudes and experiences, aligning with established research methodology standards for maintaining data quality and validity.

The researchers conducted a field study using online surveys. We sent out project information and consent forms to students through teachers’ teaching classes and student groups on social networking platforms such as Facebook and Zalo. First, the respondents were asked, “Have you ever interacted with gamification in courses?” Those who answered “yes” continued to answer the follow-up questions, whereas those who answered “no” were asked to withdraw from the survey. Thus, the respondents experienced gamification in the gamified courses. We inform the respondents of the mention of gamification in learning, including all platforms and game applications that support learning, such as Kahoot!, Slido, and Quizziz. The researchers used Google Forms to create the survey tool, and respondents were required to provide an email address to ensure its validity. We used Excel and SmartPLS 4.0, a statistical software that employs a partial least squares (PLS) approach to analyze the data.

## Ethics Statement

We value the privacy of individuals who have voluntarily participated in our research. The research team informed information related to the research to participants before taking place the survey. Our data does not reveal any identifying information about respondents because we protect their human rights. The survey is completely anonymous and does not contain any information that could identify participants.

## CRediT authorship contribution statement

**Bang Nguyen-Viet:** Writing – original draft, Writing – review & editing, Supervision. **Bac Nguyen-Viet:** Writing – original draft, Writing – review & editing. **Cuong Nguyen-Duy:** Writing – review & editing.

## Data Availability

Nguyen-Viet, Bang (2023). Dataset on the effect of gamification elements on learning effectiveness: A case study of Vietnamese students (Original data) (Mendeley Data)

## Appendix Supplementary Materials

Image, application 1
