# Supplementary material for: Dataset on the effect of gamification elements on learning effectiveness among Vietnamese students
Source: Data Brief. 2023 Oct 28;51:109734. doi: 10.1016/j.dib.2023.109734 (PMC10641147; doi:10.1016/j.dib.2023.109734)
Supplement: Supplementary file 1 [file mmc1.docx]

**QUESTIONNAIRE**

This survey focuses on examining the impact of Factors of Gamification on Effectiveness Learning. In this survey I want you to focus your thoughts on Factors of Gamification and Effectiveness Learning.There are no right or wrong answers. Please answer as honestly as you can.

**PART 1: information of respondents**

**Gender**

| Male | 0 |
| --- | --- |
| Female | 1 |

**Area of the university**

| Ha Noi | 0 |
| --- | --- |
| Ho Chi Minh | 1 |

**Age**

| From 18 years to 22 years | 0 |
| --- | --- |
| From 22 years to 26 years | 1 |
| From 26 years to 30 years | 2 |
| Over 30 years | 3 |

**Educational level**

| Student | 0 |
| --- | --- |
| MA Student | 1 |

**PART 2: Statements related to Factors of Gamification and Effectiveness Learning**

Please indicate the extent of your agreement or disagreement with each of the following statements

1. Strongly disagree 2. Disagree 3. Undecided 4. Agree 5. Strongly agree

| Statements | |  |
| --- | --- | --- |
| Competitveness (CO) | CO1: I feel competitive with other students when using gaming platforms | 1 2 3 4 5 |
|  | CO2: Gaming Platforms provide a competition system when I take a quiz | 1 2 3 4 5 |
|  | CO3: I want to get the highest rating when using the gaming platforms | 1 2 3 4 5 |
| Enjoyment (ENJ) | ENJ1: I enjoy using games in the classes | 1 2 3 4 5 |
|  | ENJ2: I feel comfortable with the game-based classes | 1 2 3 4 5 |
|  | ENJ3: I'm enthusiastic about game-based classes when carrying out the learning process | 1 2 3 4 5 |
|  | ENJ4: I feel happy when I spend time with games for learning | 1 2 3 4 5 |
| Challenge (CH) | CH1: Gaming platforms indicate the number of questions to be answered | 1 2 3 4 5 |
|  | CH2: Gaming platforms provide video or image aids to help solve questions | 1 2 3 4 5 |
|  | CH3: Gaming platforms provide a time limit for answering questions | 1 2 3 4 5 |
|  | CH4: I feel challenged when competing in the game-based classes | 1 2 3 4 5 |
| Intrinsic Motivation (IM) | IM1: I enjoyed the game-based classes very much. | 1 2 3 4 5 |
|  | IM2: Game-based classes was fun to take | 1 2 3 4 5 |
|  | IM3: I thought game-based classes was boring (reversed). | 1 2 3 4 5 |
|  | IM4: I would describe the game-based classes as very interesting. | 1 2 3 4 5 |
| Satisfaction (SA) | SA1: Overall, I am satisfied with game-based learning | 1 2 3 4 5 |
|  | SA2: Gaming platforms that I used in classes have met my expectations | 1 2 3 4 5 |
|  | SA3: I am delighted with the experience I get when using gaming platforms in classes | 1 2 3 4 5 |
| Engagement (ENG) | ENG1: Whenever I have to use games in classes, I usually use | 1 2 3 4 5 |
|  | ENG2: I am passionate about the games in classes | 1 2 3 4 5 |
|  | ENG3: I love the games in classes | 1 2 3 4 5 |
|  | ENG4: I am excited when using the games in classes | 1 2 3 4 5 |
|  | ENG5: I am proud of using the games in classes. | 1 2 3 4 5 |
| Effectiveness Learning (EP) | EP1: Game-based learning improves my grade in the class | 1 2 3 4 5 |
|  | EP2: Applying game-based learning encourages me to continue learning on the gamified platform by myself | 1 2 3 4 5 |
|  | EP3: Use of the gaming platforms have improved my overall learning performance | 1 2 3 4 5 |

THANK YOU VERY MUCH FOR YOUR TIME AND EFFORT IN COMPLETING THE QUESTIONNAIRE
